# Transcriptional Basis of Drought-Induced Susceptibility to the Rice Blast Fungus *Magnaporthe oryzae*

**DOI:** 10.3389/fpls.2016.01558

**Published:** 2016-10-27

**Authors:** Przemyslaw Bidzinski, Elsa Ballini, Aurélie Ducasse, Corinne Michel, Paola Zuluaga, Annamaria Genga, Remo Chiozzotto, Jean-Benoit Morel

**Affiliations:** ^1^INRA, UMR BGPI INRA/CIRAD/SupAgro, Campus International de BaillarguetMontpellier, France; ^2^SupAgro, UMR BGPI INRA/CIRAD/SupAgro, Campus International de BaillarguetMontpellier, France; ^3^Institute of Agricultural Biology and Biotechnology, National Research CouncilMilan, Italy

**Keywords:** stress combination, drought, fungus, immunity, effectors, rice, *Magnaporthe oryzae*

## Abstract

Plants are often facing several stresses simultaneously. Understanding how they react and the way pathogens adapt to such combinational stresses is poorly documented. Here, we developed an experimental system mimicking field intermittent drought on rice followed by inoculation by the pathogenic fungus *Magnaporthe oryzae*. This experimental system triggers an enhancement of susceptibility that could be correlated with the dampening of several aspects of plant immunity, namely the oxidative burst and the transcription of several pathogenesis-related genes. Quite strikingly, the analysis of fungal transcription by RNASeq analysis under drought reveals that the fungus is greatly modifying its virulence program: genes coding for small secreted proteins were massively repressed in droughted plants compared to unstressed ones whereas genes coding for enzymes involved in degradation of cell-wall were induced. We also show that drought can lead to the partial breakdown of several major resistance genes by affecting *R* plant gene and/or pathogen effector expression. We propose a model where a yet unknown plant signal can trigger a change in the virulence program of the pathogen to adapt to a plant host that was affected by drought prior to infection.

## Introduction

Plants are often facing several simultaneous stresses, including biotic and abiotic (Suzuki et al., 2014). The plant’s response and adaptation to such complex interactions remain largely unknown. While pathogens have constantly been a threat to plants, in particular fungi (Dean et al., 2012), drought is becoming an increasing constraint, for instance for rice cultivation (Pandey and Shukla, 2015). Drought can have positive effect and reduce disease levels but in many cases drought is increasing disease susceptibility (Kissoudis et al., 2014; Prasch and Sonnewald, 2015; Ramegowda and Senthil-Kumar, 2015). For instance, it is well-known that rainfed rice suffering for repeated and intermittent drought heavily suffers from blast disease caused by the fungus *Magnaporthe oryzae* (Bonman, 1992).

Each drought and pathogen stress are well-studied individually and many components regulating them are known. On the one hand, the plant’s response to drought has been extensively studied in model plants like *Arabidopsis* or crops like rice. The production of abscisic acid (ABA) hormone and reactive oxygen species (ROS) are among the most conserved molecular responses, together with the production of osmoprotectants and antioxidant activities (Shanker et al., 2014; Pandey and Shukla, 2015). All these changes are also associated with an intensive transcriptional re-programming (Nakashima et al., 2014). On the other hand, plant immunity is usually understood as a two-layer process (Jones and Dangl, 2006). First, transmembrane receptors recognize pathogen-associated molecular patterns and trigger basal immunity that operates as a general, non-specific immunity that reduces but does not suppress pathogen growth. Pathogens have evolved effector molecules that counteract this basal immunity and thus enhance their capacity to grow in plant tissues. These effectors are often small secreted proteins that, altogether with other enzymes like cell-wall degrading enzymes (CAZY), represent major components of the pathogen’s virulence program (King et al., 2011; Asai and Shirasu, 2015). Second, plants have developed intracellular receptors that activate the so-called effector-triggered immunity upon detection of effector(s). This mechanism, activated when a matching effector is detected by its cognate receptor, leads to complete resistance often associated with cell death localized at the site of infection. It is also known as the gene-for-gene resistance in which the receptor and the effector are, respectively, called Resistance (R) and avirulence (avr) proteins. Basal and effector-triggered immunity are often associated with the production of ROS called the oxidative burst and with the increased transcription of defense-related genes like pathogenesis-related (*PR*) genes (Jones and Dangl, 2006). While several reports (see below) indicate that basal immunity pathway is connected to the drought tolerance pathway, to our knowledge there is yet no report that drought can impact effector-triggered immunity.

The relationships between drought and disease tolerance/resistance pathways can be estimated by the analysis of transgenic or mutant plants. Quite strikingly, the analysis of the majority of transgenic plants showed that drought tolerance and disease resistance pathways are often antagonist (Delteil et al., 2010). For instance, the over-expression of the major regulator of disease resistance OsNPR1 increased disease resistance but reduced drought tolerance in rice (Quilis et al., 2008). Conversely, silencing of OsMPK5 enhanced drought tolerance but reduced disease resistance (Xiong and Yang, 2003). Most of these antagonistic effects have been attributed to the activation of the ABA pathway under drought stress which is thought to be detrimental to the setting of disease resistance pathways (De Vleesschauwer et al., 2014). For all these reasons, one hypothesis to be tested is that basal immunity is repressed by drought signaling.

Indeed, the way plant immunity is deployed after drought is not well characterized and only very few studies have been published. In a pioneer study, it was shown that most of the plant reaction to a combined biotic and abiotic stress could not be predicted from the knowledge of the individual stress (Rasmussen et al., 2013) but this work did not involve drought nor an entire pathogen. Soon after this work, two categories of experiments were conducted: drought followed by infection or infection and then drought. In their study, Atkinson et al. (2013) first inoculated *Arabidopsis* with the nematode *Heterodera schachtii* and then imposed continuous drought. Overall, the drought-response pathway was dominating as evidenced by the fact that most of transcriptional changes were related to this stress and less to disease-related stress. Similarly, the analysis of viral infection by TuMV followed by drought showed that drought abolished most of the *Arabidopsis* genes known to respond to this virus (Prasch and Sonnewald, 2013). On the other hand, when the bacteria *Xylella fastidiosa* was inoculated to grape *after* drought, it was observed that the increased symptoms to the pathogen associated with an enhanced drought response, with no indication that the activation of the plant’s immune system was strongly affected (Choi et al., 2013). In the case of the inoculation by the virulent bacteria *Pseudomonas syringae* pv. *tomato* in *Arabidopsis* plants grown under continuous drought, it was found that reduced susceptibility could be correlated to an increased expression of basal immunity genes (Gupta et al., 2016). Similar results were obtained by inoculating *P. syringae* pv. *tabaci* and the necrotrophic fungus *Sclerotinia sclerotiorum* on tobacco plants acclimated to drought stress (Ramegowda et al., 2013). The impact of an abiotic stress on molecular components of pathogen virulence has also been scarcely studied. For instance, pathogen adaptation to abiotic stress involves molecules with pleiotropic effect that are considered to be virulence factors like siderophores (Hof et al., 2007) and temperature altered the expression of several bacterial effectors [Cheng et al. (2013) and references therein]. Thus there are only very few examples documenting the molecular impacts of drought on disease.

In this study, we used the model interaction between rice and the devastating blast fungus *M.* oryzae to address several questions relative to the impact of drought on plant/fungal interactions. After developing a protocol where an intermittent mild drought stress severely increased blast susceptibility, we looked how basal immunity was impacted by drought in the early times of infection. Using RNASeq analysis, we also tested if the fungal pathogenicity program was affected by drought applied prior to infection. This led us to show that drought can also affect effector-triggered immunity. We propose a model where an unknown plant signal indicative of the plant physiological status is likely sensed by the pathogen that adapts its invasion strategy accordingly.

## Materials and Methods

### Plant Growth and Drought Stress Protocol

Indicated rice genotypes (*Oryza sativa*) were sown on Thursdays (day 1) in Neuhaus S soil mixed with poudzolane (2L/70L). Each replicate consisted of eight plants grown in 5 cm × 5 cm × 5 cm pot (hxlxl) at 29°C day/21°C night, 16 h light regime. Plants were watered daily and fertilized on Mondays, starting from the second Monday after sowing. At day 19, trays were inundated with ∼2 cm water level. At day 23 (Friday; 14:00 h), drought stress was imposed by removing water. Three days later (Monday; day 26; 11:00 h), drought stressed plants were put back on the tables allowing rehydration for 2 h followed by spray inoculation. Field capacity was measured by DW/WW × 100 where WW and DW is wet and dry soil weights, respectively. The 3 and 4 days drought corresponded to 50 and 38% field capacity, respectively, while control plants were maintained at 100%. These values were similar to those used in a similar study (Gupta et al., 2016).

### Inoculation and Disease Symptoms Analysis

Indicated *M. oryzae* isolates were cultured as in Berruyer et al. (2003). Spores were collected by scraping and rinsing plates filled with distilled water. After filtering through two layers of nylon mesh, spores were counted in hemocytometer. Finally, inoculum was prepared in water containing 0.5% gelatine. Plants were inoculated by spraying 30 mL of inoculums (10^6^ conidia mL^-1^) on 24 pot trays. Control plants were sprayed with 0.5% solution of gelatin (mock treatment). Volumes were adjusted proportionally to the number of pots sprayed. Inoculated plants were placed for 16 h in a climatic chamber set at 25°C with the humidifiers working in 15 min cycles to saturate the air with water. The plants were then returned to normal growth conditions. Disease symptoms were recorded at 7 days after inoculation.

### Transgenic Isolates

The multi-virulent isolate GUY11 was transformed with plasmids carrying the avirulence genes *Avr-Pia* or *Avr-Pii* (Yoshida et al., 2009). The plasmid for generating transgenic *M. oryzae* carrying *Avr-Pita* was created by PCR-amplifying the *Avr-Pita* CDS as well as 489 bp promoter sequence from pCB980 (Orbach et al., 2000) (forward oTK097 CTATAGGGCGAATTGGGTACTCAAATTGGTTGCCGAGTCGTTCTGAGGG, reverse oTK098 GGAGCCTGAATGTTGAGTGGAATGATCCCTCTATTGTTAGATTGACC).

### Cytological Analysis

Tissue staining and observations for disease progression analysis and H_2_O_2_ accumulation were done as previously described in Ballini et al. (2013) and in Faivre-Rampant et al. (2008), respectively.

### RNA Extraction for RT-qPCR and RNAseq

Gene expression analysis including RNA extraction and RT-qPCR was performed as previously described in Delteil et al. (2012). The primers used in this study are given in **Supplementary Table S1**. Each replicate was composed of six plants. Four replicates were used if not stated differently. Briefly, we used LC480 SYBR Green I Master Mix (Roche, Basel, Switzerland). Amplification on LightCycler 480 thermocycler (Roche) was as follows: 95°C for 10 min; 40 cycles of 95°C for 15 s, 60°C for 20 s, and 72°C for 30 s; then 95°C for 5 min and 40°C for 30 s. RNA for high throughput sequencing was additionally purified using chloroform. RNA integrity of the samples was assessed with Bioanalyzer 2100 (Agilent) and only samples with RIN at least 7.5 were used. High throughput RNA sequencing was performed by BGI Tech (Shenzhen, China).

### RNAseq Data Analysis

Initial data cleaning and reads mapping was performed by BGI Tech (Shenzhen, China). The libraries were sequenced as 50-bp single-end reads using Illumina Hiseq2000 according to the manufacturer’s instructions. Clean reads have been mapped using SOAP2.21 on reference genomes: Nipponbare for rice and 70-15 for *Magnaporthe*. Annotated rice transcripts from Nipponbare genome were provided by BGI Tech. *M. oryzae* annotation datasets were downloaded from the *Magnaporthe* comparative Sequencing Project at the Broad Institute (assembly release 8) (Chiapello et al., *2015*). We chose 4 dpi for RNASeq analysis as in our experience, fungal RNA is too diluted in earlier time points for appropriate detection and statistical analysis (Kawahara et al., 2012). For one repetition and on average, 528 k reads were found with a unique perfect match on *M. oryzae* Mg8. The enrichment in *M. oryzae* was corrected by normalization procedures in order to detect differentially expressed genes between the two conditions (drought-stressed and unstressed). A total of 9388 genes were kept for DEG analysis because they were considered as expressed at 4 dpi, i.e., when the sum of read counts for the three repetitions of one of the condition was >5. For rice, on average 40 million reads with perfect match were mapped on the genome. Twenty-nine thousand five hundred and seventy-five genes with at least 40% coverage were kept for the differential gene expression (DEG) analysis. The DEG analysis was performed on DEB website (Yao and Yu, 2011). For DEG analysis of *M. oryzae* genes, we used two different statistical analysis methods, namely DESeq and EdgeR with a minimum FDR of 5%. We considered as differentially expressed all genes that were significant in at least one of the statistical test. Enrichment analyses of the different functions were tested against the whole genome annotation with a χ^2^ test corrected by Bonferroni. For DEG analysis of rice genes, we considered as differentially expressed genes, one that were significant (FDR of 5%) in at least one of the conditions. Difference in log2 fold change ratio between drought-stressed and unstressed conditions were tested using agricolae R package with a Kruskal–Wallis test corrected by Bonferroni with a *p*-value of 1%. Bam files, reads counts and RPKM per gene are available on GEO database (GSE84800).

### Annotation of *M. oryzae*

The reference genome Mg8 was used for the analysis. This annotation is based on the genome sequence of the 70-15 isolate and we used a closely related isolate FR13 (Chiapello et al., 2015). Gene coding for Carbohydrate-Active enZYmes (CAZY) were annotated based on CAZY.org database (Lombard et al., 2014). Small secreted proteins were annotated based on three criteria: (1) the presence of a signal peptide longer than 15 aa at the beginning of the gene, (2) the absence of a transmembrane (TM) domain or a TM domain in the first 10 aa, and (3) a protein size smaller than 250 aa. Signal peptide was detected using SignalP software (Petersen et al., 2011). Transmembrane domains were detected using TMHMM program (Krogh et al., 2001).

### Annotation of Rice

Complementary annotations were made for gene function and gene expression in stress conditions. Pathogenesis related proteins (PR) were annotated based on literature and function annotation for the 17th PR families. Defense regulators were annotated based on literature (Vergne et al., 2008). Gene expressed in drought stress were annotated based on literature on rice microarray under this stress (Rabbani et al., 2003; Zhang et al., 2012; Ding et al., 2013; Narsai et al., 2013).

## Results

### Controlled, Mild Drought Stress is Strongly Increasing Susceptibility to Rice Blast

**FIGURE 1:**
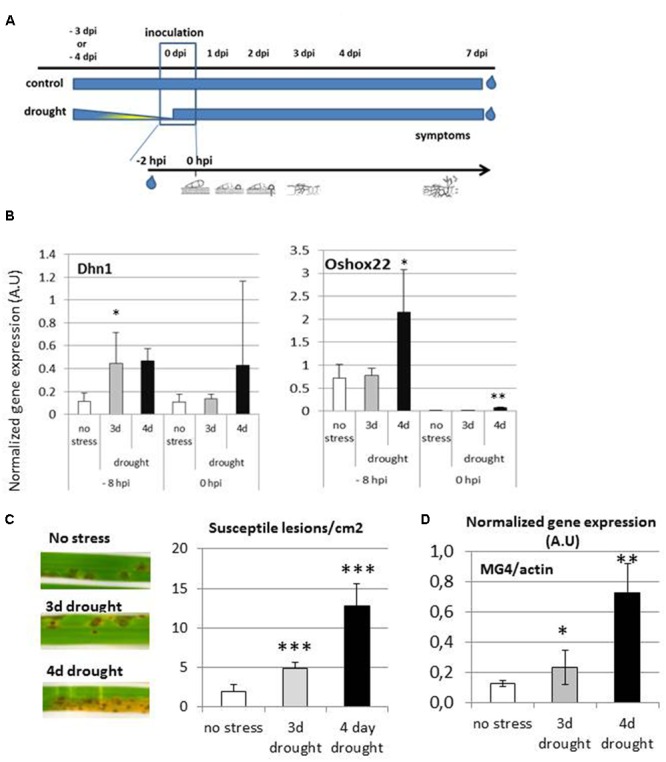
**Rice infection by *Magnaporthe oryzae* after drought stress.** Three weeks old rice plants were subjected to drought stress and then spray inoculated with *M. oryzae* spores (FR13 isolate). Details of drought treatment, rehydration, inoculation, and sampling are schematically presented in panel **(A)**. **(B)** Expression of drought-inducible Dhn1 and Oshox22 marker genes before fungal inoculation. The expression was measured before re-watering (-8 hpi) and 2 h after re-watering (at the time of inoculation, 0 hpi). Gene expression was measured by RT-qPCR and normalized with the Actin gene (see Materials and Methods). **(C)** Macroscopic disease symptoms at 7 days after inoculation and the number of lesions per leaf surface. **(D)** Quantification of the disease symptoms presented in **(C)** using Q-PCR to measure fungal biomass (MG4 constitutive fungal gene) normalized with the constitutive Actin rice gene. Asterisks indicate significant differences to the well-watered control plants in the *t*-test (^∗∗∗^*P* < 0.001, ^∗∗^*P* < 0.01, ^∗^*P* < 0.05).

It was previously documented that rice blast disease is more severe after a period of drought (Bonman, 1992; Bocco et al., 2012). To better understand this phenomenon and to be able to characterize it at the molecular level, we decided to establish a controlled-drought experiment followed by inoculation of the plants (**Figure 1A**). We tested two conditions of imposed drought length after which plants were re-watered. Three and four days of progressive dehydration resulted, respectively, in mild to strong leaf rolling and pots lost 40 and 60% of their initial weigh due to water evaporation (**Supplementary Figure S1A**). Leaf surface or plant height of 3-day stressed plants was not affected and was slightly decreased after 4-day drought (**Supplementary Figures S1B,C**). After drought and before re-watering, the expected induction of drought-responsive rice genes indicated that the plants were under stress in both 3- and 4-day treatments (see -8 hpi time point in **Figure 1B** and **Supplementary Figure S1D**). After re-watering but before inoculation (see 0 hpi time point in **Figure 1B** and **Supplementary Figure S1D**), the expression of most of drought-inducible gene markers was identical to non-stressed plants in 3 days-stressed plants but remained high in 4 days-stressed plants, suggesting that the 4-days drought induced a stronger stress than the 3-days one, consistent with stronger impact on plant development (**Supplementary Figures S1B,C**).

Two hours after re-watering, plants were inoculated with the FR13 isolate of *M. oryzae* which is fully virulent on the reference, Nipponbare variety (Delteil et al., 2012). Plants subjected to 3-day drought stress developed more disease symptoms than unstressed plants (**Figure 1C**). Leaves of 4-day stressed plants presented even more severe disease symptoms, with additional grayish zones corresponding to sporulating colonies. This enhanced susceptibility was further confirmed by quantification of fungal abundance by qPCR (**Figure 1D**), with 4-day stressed plants showing more fungal DNA than 3-day stressed. Because 3-day drought stress significantly increased disease susceptibility and the 4-day stress resulted in plant development reduction, we decided to focus on the milder drought stress for further analyses. Detailed cytological analysis of disease progression revealed no difference at early stages of the infection (before 40 hpi; **Supplementary Figure S1E**) and enhanced fungal growth was measured afterward, consistent with the increase of the symptoms.

We further evaluated the impact of drought on rice blast susceptibility on indica and japonica rice accessions and with a broadly virulent isolate, Guy11. All rice accessions showed an enhancement of susceptibility symptoms (**Supplementary Figure S2**), showing that our experimental protocol generally affects rice blast susceptibility. Taken together, the mild drought stress imposed here before inoculation results in much higher disease susceptibility of rice plants to *M. oryzae* while it does not significantly affect plant development. This experimental design was used to better understand how plant is mounting defense after a drought period and how the fungus reacts to this new cellular environment.

### Drought Stress Affects *M. oryzae* Virulence Program

**FIGURE 2:**
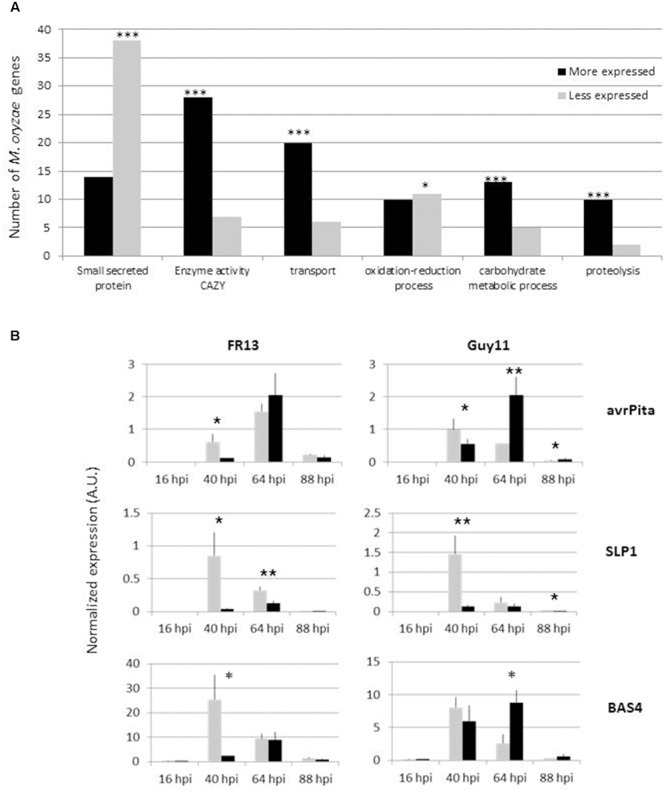
**Impact of drought on transcriptional regulation of pathogenicity processes.** Nipponbare plants were drought-treated (3-day drought) or not and then infected with *M. oryzae* FR13 isolate according to the protocol described in **Figure 1**. RNAseq analysis at 4 dpi was used to measure fungal gene expression *in planta*. **(A)** The fungal genes differentially expressed (more or less after droughted compared to unstressed plants) were categorized (see Materials and Methods) and the frequency of each category was compared to the frequency expected from the fungal genome. Only the categories with more than 10 genes are shown. A χ^2^ test (corrected by Benjamini) was used to evaluate significant differences between dataset and genome (^∗^*P* < 0.05; ^∗∗∗^*P* < 0.001). **(B)** Gene expression of three fungal effectors was measured by RT-qPCR during all fungal cycle in plants that experienced no stress (gray) or 3-day drought (black bars). Two different virulent isolates of *M. oryzae* were used (Guy11 and FR13). The expression was normalized with the constitutive fungal gene MG4. The data represent the mean and SD from four replicates. A *t*-test was used to compare gene expression in non-stressed and drought stressed plants (^∗∗^*P* < 0.01; ^∗^*P* < 0.05).

RNA sequencing was used at 4 dpi to monitor fungal gene expression in drought-stressed and unstressed plants. The RNA sequencing depth (see Materials and Methods) allowed a good coverage of *M. oryzae* genes (in average 38% gene coverage). A total of 336 fungal genes were found as differentially expressed in at least one of the statistical analysis (**Supplementary Table S2**). Based on our annotation analysis in this gene set, 52 genes are putative small secreted protein and 35 are cell-wall degrading, CAZY enzymes (respectively, 15.5 and 11.3% of DEG genes). In the 12827 genes that, we annotated in our database, only 800 have been identified as putative small secreted protein (*i.e.*, 6%) and 482 as CAZY enzyme (*i.e.*, 3.8%). Thus the DEG set is significantly enriched in both protein categories (**Figure 2A**) that are major players in fungal virulence. When, we then looked at the expression values, a clear picture was visible: genes coding for putative effectors were repressed while those for CAZY enzymes were induced in drought-stressed plants compared to unstressed ones (**Figure 2A**). We further extended this observation by measuring with RT-qPCR fungal gene expression in two different *M. oryzae* strains at different time points along the infection process. These analyses showed that the expression of most tested fungal effectors (*Avr-Pita, BAS1, BAS4, SLP1, SLP2*; **Figure 2B** and **Supplementary Figure S3**) was reduced in drought-stressed plants compared to unstressed ones. For Guy11, a lesser reduction of expression was also accompanied with a delay in expression. Moreover, out of the 112 *M. oryzae* genes repressed, 38 are putative effectors (34%) while only 6% would have been expected from genomic data (**Supplementary Table S2**). Thus the drought stressed first imposed to plants strongly affected the expression of two major categories of *M. oryzae* genes involved in virulence.

### Basal Plant Immunity is Severely Reduced after Drought Stress

**FIGURE 3:**
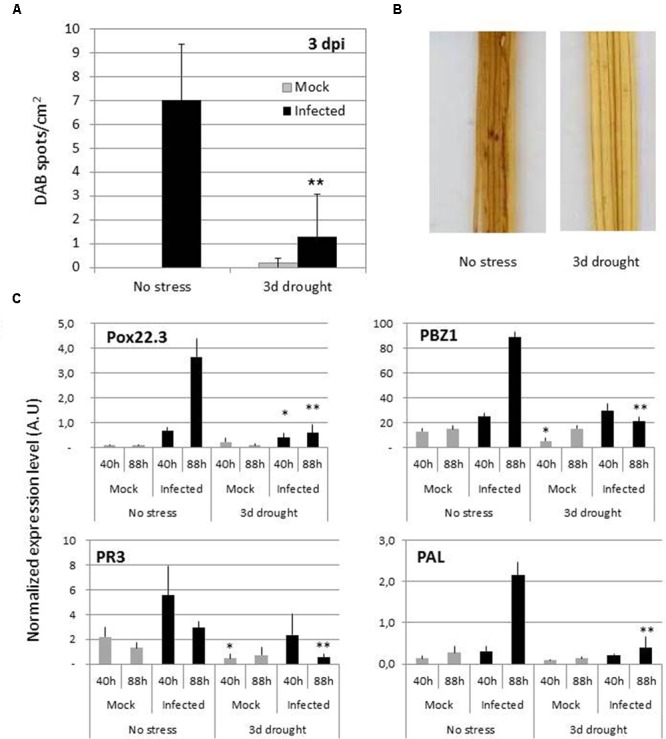
**Oxidative burst and transcriptional response of rice to *M. oryzae* infection after drought.** The oxidative burst **(A,B)** and the expression of defense-related genes **(C)** was measured in Nipponbare plants inoculated as in **Figure 1** after 3-days drought. Plants were spray inoculated with *M. oryzae* fungus (Guy11) or mock treated (0.5% gelatine). **(A,B)** DAB staining was used to visualize oxidative burst in plants at 3 dpi. A *t*-test was used to compare gene expression in non-stressed and drought stressed plants (^∗∗^*P* < 0.01). **(C)** Expression of the disease responsive marker genes was measured by RT-qPCR and normalized to rice Actin. The values are the mean and SD from four replicates. For each time point, a *t*-test was used to compare gene expression in non-stressed and drought stressed plants (^∗∗^*P* < 0.01;^∗^*P* < 0.05).

Two major facets of plant immunity, namely the production of ROS during the oxidative burst and the induction of defense genes, were measured after infection. Three days after infection, when differences can be observed for fungal growth (**Figure 1D** and **Supplementary Figure S1E**), ROS production was detected in infected plants (but not mock treated ones) and this production was severely reduced in drought-stressed plants (**Figures 3A,B**).

At the time of inoculation in 3 days droughted plants, we observed no strong modification of the defense-inducible plant genes tested (Delteil et al., 2012) (**Supplementary Figure S1D**). After infection, these four markers of plant defense were induced in unstressed plants but this induction was strongly reduced in drought-stressed plants (**Figure 3C**). The 4 dpi time point used for RNASeq analysis of *M. oryzae* gene expression was also used to monitor plant gene expression (**Supplementary Figure S4**; **Supplementary Table S3**). Plant DEGs specific to infection were the most numerous (6625) compared to those specific to drought (119), suggesting that plants are responding to infection rather than to drought at this time point. This is also likely to relate to the fact that at the time of our analysis, drought has been stopped for more than 4 days. General stress-related plant genes had an expression enhanced by the combination of stresses (**Supplementary Figure S4C**) and the expression pattern of 503 genes could not be predicted from single stress situations (**Supplementary Figure S4D**). Interestingly 114 plant genes are part of a gene set with an expression antagonistically modified by drought and infection. This gene set is enriched in defense-related genes. For example PBZ1 and POX22.3 were found in the category of DEGs that had an expression pattern canceled by drought (**Supplementary Figure S4E**). Altogether, these RNASeq data indicate that plant immunity at late time points after inoculation is lower in drought-stressed than in non-stressed plants.

### Drought Provokes the Partial Breakdown of Several Major Resistance Genes

**FIGURE 4:**
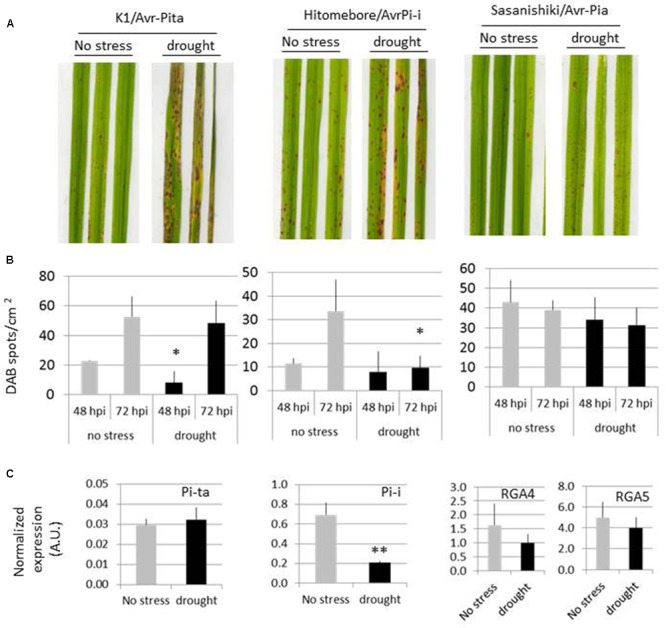
**Analysis of R gene-mediated resistance after drought.** The indicated rice genotypes were grown, drought-treated and inoculated as presented in **Figure 1**. Plants were spray inoculated with *M. oryzae* Guy11 isolate complemented with the indicated *avr* genes under their native promoter. **(A)** Macroscopic phenotypes were analyzed at 7 days after inoculation. **(B)** The oxidative burst was measured using DAB staining at the indicated time after inoculation. For each time point, a *t*-test was used to compare ROS production in non-stressed and drought stressed plants (^∗^*P* < 0.05). **(C)** At the time of inoculation, the expression of the immune receptor recognizing the corresponding avr protein was measured by RT-qPCR and normalized with the rice Actin gene. For Pi-a, two immune receptors are involved (Cesari et al., 2013). A *t*-test was used to compare gene expression in non-stressed and drought stressed plants (^∗∗^*P* < 0.01).

Effectors like Avr-Pita can be recognized by their cognate resistance protein in the plant. This recognition triggers cell death and complete plant resistance. Based on the observation that many effectors were down-regulated (**Figure 2**) and that plant immunity was overall depressed in drought-stressed plants (**Figure 3**), we asked whether major resistance genes were still operating under such conditions. We tested three major resistance genes (*Pi-ta, Pi-i*, and *Pi-a*) by using Guy11 transgenic strains expressing the corresponding effectors expressed under their own promoter. The FR13 strain could not be used as it already contains several AVR genes. The *Pi-ta* and the *Pi-i* genes showed a partial breakdown of resistance, with the development disease symptoms, while *Pia* plants remained resistant (**Figure 4A**). This correlated with reduced ROS production in *Pi-ta* and *Pi-i* plants (**Figure 4B**). In addition, the *Pi-i* gene showed reduced transcription in drought-stressed plants whereas the expression of *Pi-ta* and the *RGA4/RGA5* genes required for Pi-a resistance remained unchanged (**Figure 4C**). Thus complete resistance can be strongly affected by drought with various degrees.

## Discussion

Drought in the field is negatively impacting rice resistance to the blast fungus *M. oryzae* as it increases lesion number, lesion size, and disease severity (Gill and Bonman, 1988). For instance, the blast infection efficiencies of plants that were subjected to severe water deficit was more than four times those of unstressed controls (Gill and Bonman, 1988). We set out an experimental system displaying a mild and intermittent drought-stress before inoculation (**Figure 1**) to identify the transcriptional and cellular mechanisms underlying such enhanced susceptibility in plants and pathogen. Moderate drought (3 days) preceding inoculation only slightly induced plant’s response to drought but greatly increased disease severity (**Figure 1C**; up to 6.5-fold) and we propose to call this phenomenon drought-induced susceptibility (DIS). The enhanced susceptibility observed in our experiments is unlikely due to an indirect effect of altered plant growth since in the 3 days stress, we could not measure any impact of drought on development (**Supplementary Figures S1B,C**). Our DIS protocol (**Figure 1A**) is likely mimicking natural situations, in particular rainfed environments with intermittent drought stress where blast disease is severe (Gill and Bonman, 1988). In such environment, rain is ending a period of drought, temporarily increasing humidity that is favorable to *M. oryzae* germination. Evaluating rice panels characterized for drought tolerance [*e.g.*, (Luo, 2010)] is now amenable to see how drought-regulation and DIS are genetically linked. Similarly, it will be interesting to evaluate if our experimental system enhances disease to other rice pathogens.

Our analysis indicates that the induction of basal immunity is dampened in plants that were pre-exposed to drought (**Figures 1B** and **3**; **Supplementary Figures S1D** and **4E**). Although this was largely expected given the well-known negative cross-talks between the pathway controlling drought response and pathogen response (De Vleesschauwer et al., 2014), this is to our knowledge the first report documenting at the transcriptional levels the impact of drought on plant’s response to fungal attack. In their study combining continuous drought followed by bacterial infection, an enhancement of basal immunity was observed, consistent with reduced susceptibility (Gupta et al., 2016), while in our study, we observe an increase of susceptibility. The difference in the way drought way applied [intermittent here and continuous in Gupta et al. (2016)] could explain such opposite consequences. The effects of drought on rice have been reviewed (Pandey and Shukla, 2015) and include an increase of ABA and several antioxidant enzymatic activities. Intermittent drought stress also caused an increase in the ABA level that rapidly returned to normal upon re-watering (Muthurajan et al., 2010; Xu et al., 2010). ABA often increases pre-penetration defenses, in particular stomatal closure, that reduce pathogen penetration (Ramegowda and Senthil-Kumar, 2015). This is unlikely operating in the case of rice blast fungus since this pathogen does not require stomata for penetration. By contrast, an inhibition of plant defense by the activation of the ABA pathway in response to drought (De Vleesschauwer et al., 2014) could explain our observation that plant immunity is down-regulated in our experimental system. Moreover, an increase of antioxidant activities during drought recovery may lead to a reduction of the pathogen-induced oxidative burst during this phase (**Figures 3A,B**). Inhibition of plant’s basal immunity is likely compensating the down-regulation of pathogen effectors’ expression (**Figure 2**; see below). Our experimental system now opens the possibility to scrutinize in more detail basal immunity to identify where and when drought is negatively impacting its onset.

It is well known that *R* gene function can be inhibited by heat stress (Gijzen et al., 1996; Wang et al., 2009; Cheng et al., 2013; Hua, 2013). By contrast, we have shown that the function of several rice *R* genes was not broken down upon another abiotic-related stress, nitrogen fertilization (Ballini et al., 2013). The observation that many fungal effectors were down regulated in plants after drought (**Figure 2**) prompted us to test the possibility of an inhibition of effector-triggered immunity by drought. Our finding that drought can affect some, but not all tested plant *R* genes extends the vulnerability of *R* genes to this abiotic stress (**Figure 3**). In the case of heat stress, it was shown that R protein re-localization is responsible for the loss of activity of these proteins (Zhu et al., 2010). Whether drought also affects R protein localization requires further analysis and several scenarios could explain the partial breakdown of resistance in our experiments: low expression of the effector gene and normal expression of the cognate *R* gene (e.g., Avr-Pita/Pi-ta) or down-regulation of the *R* gene (e.g., *Pi-i*). Our dataset did not allow following the global expression of *R* gene analogs during drought because, we measured the expression 4 days after the start of drought recovery. We used the RiceDB database (Narsai et al., 2013) to retrieve rice *R* gene analogs differentially expressed in drought experiments and found out that 26 are repressed and 11 are induced. Thus some *R* gene analogs are regulated and often repressed by drought. ABA could also be responsible for part of the observed partial breakdown of resistance as it was shown to inhibit effector-triggered immunity (McDonald and Cahill, 1999; Cao et al., 2011; Kim, 2012).

**FIGURE 5:**
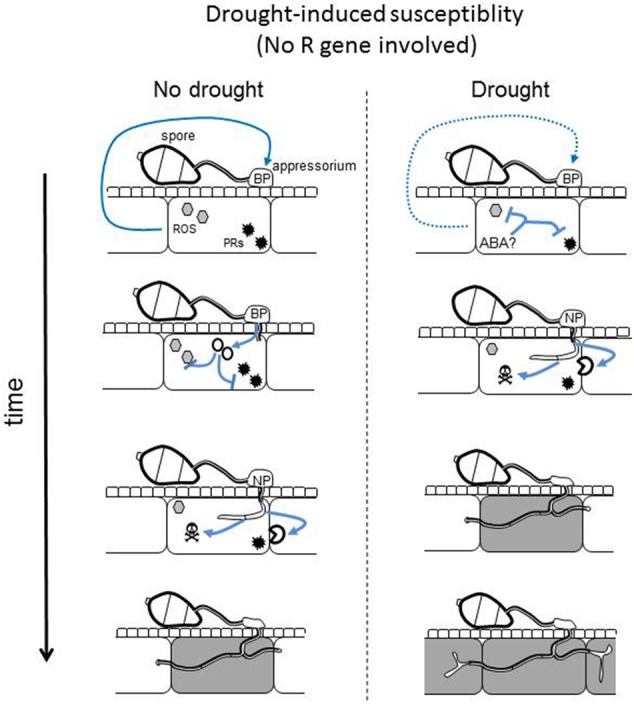
**Hypothetical model for drought-induced susceptibility.** Under non-stressed conditions, an unknown plant signal informs the fungus on the level of plant’s immunity (blue arrow). This signal activates the fungal pathogenicity program associated with biotrophy (BP): effectors are produced (red circles) to inhibit plant immune response including ROS (gray hexagons) and defense proteins (black stars). Once immunity inhibited and biotrophy established, a nectrotrophy program (NP) is activated that produces cell wall degrading enzyme and other toxin activities, leading to plant cell death (gray cell) and pathogen facilitated growth. After the response to drought, some plant signals like ABA and the production of antioxidant activities might inhibit the production of the immune response. The unknown plant signal indicative of high immunity is weaker or no longer present (dashed arrow). In the absence of this signal, the fungus is rapidly activating its necrotrophic program.

Our results suggest that the observed differences in blast susceptibility between plants under different watering treatments were not due to the differences in the pre-penetration activity of *M. oryzae*, consistent with previous findings (Kim, 1984). We thus looked for transcriptional changes in the fungus after penetration to look how the fungus’ biology was modified by plant differential treatments. For the first time a differential expression analysis was conducted on *Magnaporthe* by RNAseq *in planta*. This analysis revealed that several functions of the pathogen are strongly impacted by the abiotic stress applied to plants before infection. Indeed, we could observe that many fungal genes coding for biotrophic effectors and cell-wall degrading enzymes are expressed to lower and higher levels, respectively, in droughted plants. This is reminiscent of the observation that bacterial effectors are expressed to low levels at high temperature, a condition also known to reduce effector-triggered immunity (Cheng et al., 2013). Thus pathogens may adapt their virulence strategy depending on the physiological status of the plant. The content in several molecular signals like amino acids, sugars and hormones known to be modified in leaves after drought (Shanker et al., 2014; Pandey and Shukla, 2015) could be perceived by the fungus. For instance, ABA and cytokinins are known to increase in leaves after drought and *M. oryzae* is known to react to these plant hormones (Spence and Bais, 2015; Chanclud et al., 2016). Similarly, *M. oryzae* can sense ROS and osmotic changes (Zhang et al., 2010) or amino acids and sugars (Wilson et al., 2007). We propose a model for the enhancement of susceptibility after an intermittent drought where immunity is low after inoculation and where pathogenicity functions are differentially expressed in drought-stressed plants compared to normally watered ones (**Figure 5**). In this model, we propose that the pathogen adapts its virulence strategy depending on one or several plant-derived signals. This strategy also favors effector-immunity breakdown by reducing the expression of effectors and/or by down-regulating *R* gene expression. The signals behind this adaptation remain to be identified and our experimental system opens the door to its identification.

## Author Contributions

PB, CM, PZ, and J-BM took care of the plants, inoculation, disease symptoms analysis and cytology. PB, CM, and AD performed RNA extractions and RT-QPCRs. EB performed the statistical analyses. EB and RC analyzed RNAseq gene expression experiments together with J-BM and PB. AG expertized the data for drought genes. J-BM and PB designed the experiments. PB drafted the manuscript. J-BM and EB completed the draft. All authors read and approved the final manuscript.

## Conflict of Interest Statement

The authors declare that the research was conducted in the absence of any commercial or financial relationships that could be construed as a potential conflict of interest.

## Supplementary Material

FIGURE S1 **Impact of drought stress on plant development, gene expression and fungal growth.** Three weeks old rice plants (Nipponbare) were subjected to 3 or 4 days progressive dehydration as in **Figure 1**. **(A)** Total weight of the pots with soil was recorded at the beginning and at the end of the dehydration and just before inoculation (0 dpi). At the end of the dehydration, plants were re-watered and their growth was evaluated 7 days later in the absence of fungal infection. The impact of the drought stress on leaf surface **(B)** and plant height **(C)** were measured. The mean and standard deviation of four replicates (each replicate corresponds to the 4 pots unit and 24 plants in total) is indicated. A *t*-test was used to compare non-stressed and drought stressed plants and different letters indicate significant differences (*P* < 0.01). **(D)** Expression of drought-inducible genes (DREB2A and NAC6) and disease-inducible (PR3, PAL, PBZ1, and POX22.3) marker genes before fungal inoculation (**Supplementary Table S1**). The expression was measured before re-watering (-8 hpi) and 2 h after re-watering (at the time of inoculation, 0 hpi). Gene expression was measured by RT-qPCR and normalized with the Actin gene (see Materials and Methods). **(E)** Cytological quantification of *M. oryzae* FR13 disease progression on Nipponbare seedlings. For simplification only advanced infection of at least two cells is presented as a percentage of all the spores observed. Each data point is an average and SD from three replicates. For each replicate, second leaves were collected from three plantlets and at least 100 infection events were counted. A *t*-test was used to compare non-stressed and 3-day stressed (^∗^*P* < 0.05; ^∗∗^*P* < 0.01).

FIGURE S2 **Drought-induced susceptibility in different rice accessions.** All plants were grown as in **Figure 1** (3 days drought then re-watering) and inoculated with the Guy11 or the CL367 isolate of *M. oryzae.* Symptoms were taken at 7 dpi. The number of grayish lesions is indicative of the susceptibility level.

FIGURE S3 ***In planta* expression of various fungal pathogenicity genes after drought.** Gene expression of two biotrophic effectors fungal effectors (SLP2, BAS1), one necrotrophic effector (NEP1) and of the pathogenicity regulator MSP1 was measured by RT-qPCR during all fungal cycle in plants that experienced no stress (gray) or 3-day drought (black bars). The expression was normalized with the constitutive fungal gene MG4. The data represent the mean and SD from four replicates. A *t*-test was used to compare gene expression in non-stressed and 3-day stressed plants (^∗∗^*P* < 0.01; ^∗^*P* < 0.05).

FIGURE S4 **Plant gene expression after inoculation and drought stress.** Rice gene expression was measured by RNAseq (see Materials and Methods) at 4 dpi in Nipponbare plants infected with the *M. oryzae* FR13 isolate. DEG analysis allowed the identification of several expression patterns and the five major ones are shown: drought-specific (D), *M. oryzae* specific (Mo), showing enhanced regulation (enh), non-predictable expression (not pred) and canceled (Canc). Each pattern could be subdivided depending on the induction (I) or repression (R) of the corresponding genes. For each group of genes, the average and SD values (after normalization) are shown. The letters above each condition reflect the statistics of each individual gene in each group. The number of genes in each pattern is indicated between brackets. **(A)** genes regulated by drought only, **(B)** genes regulated by infection only, **(C)** genes whose induction by infection is enhanced by drought, **(D)** genes whose expression cannot be predicted from single stresses, and **(E)** genes whose regulation by infection is cancelled by drought.

TABLE S1 **Primers used for RT-qPCR**.

TABLE S2 ***Magnaporthe oryzae* gene differentially expressed in drought-stressed plants compared to unstressed plants**.

TABLE S3 **Rice genes showing dual effect of drought and infection**.
