# Supplementary figures and images for: Transcriptional Basis of Drought-Induced Susceptibility to the Rice Blast Fungus Magnaporthe oryzae
Source: Front Plant Sci. 2016 Oct 27;7:1558. doi: 10.3389/fpls.2016.01558 (PMC5081564; doi:10.3389/fpls.2016.01558)

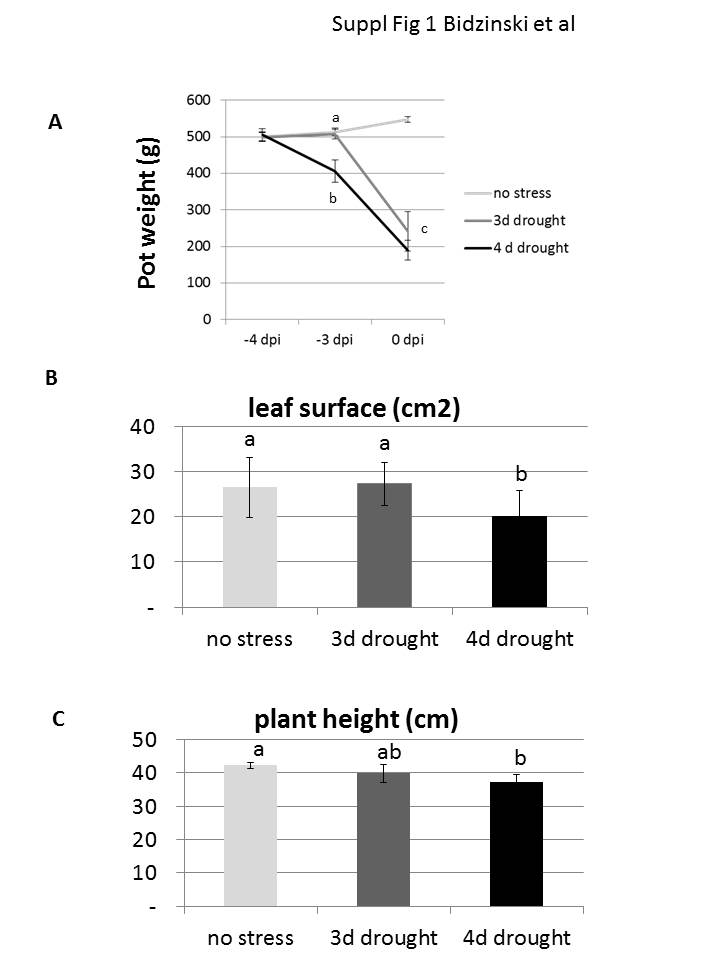

Supplement: FIGURE S1 — Impact of drought stress on plant development, gene expression and fungal growth. Three weeks old rice plants (Nipponbare) were subjected to 3 or 4 days progressive dehydration as in Figure 1. (A) Total weight of the pots with soil was recorded at the beginning and at the end of the dehydration and just before inoculation (0 dpi). At the end of the dehydration, plants were re-watered and their growth was evaluated 7 days later in the absence of fungal infection. The impact of the drought stress on leaf surface (B) and plant height (C) were measured. The mean and standard deviation of four replicates (each replicate corresponds to the 4 pots unit and 24 plants in total) is indicated. A t-test was used to compare non-stressed and drought stressed plants and different letters indicate significant differences (P < 0.01). (D) Expression of drought-inducible genes (DREB2A and NAC6) and disease-inducible (PR3, PAL, PBZ1, and POX22.3) marker genes before fungal inoculation (Supplementary Table S1). The expression was measured before re-watering (-8 hpi) and 2 h after re-watering (at the time of inoculation, 0 hpi). Gene expression was measured by RT-qPCR and normalized with the Actin gene (see Materials and Methods). (E) Cytological quantification of M. oryzae FR13 disease progression on Nipponbare seedlings. For simplification only advanced infection of at least two cells is presented as a percentage of all the spores observed. Each data point is an average and SD from three replicates. For each replicate, second leaves were collected from three plantlets and at least 100 infection events were counted. A t-test was used to compare non-stressed and 3-day stressed (∗P < 0.05; ∗∗P < 0.01). [file Image_1.JPEG]

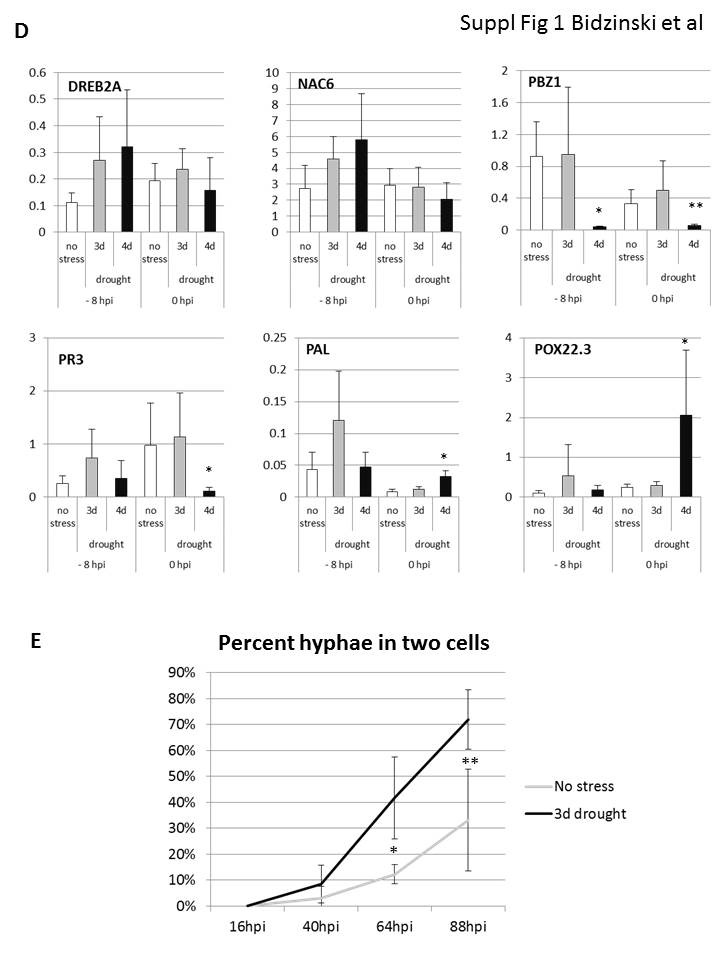

Supplement: FIGURE S2 — Drought-induced susceptibility in different rice accessions. All plants were grown as in Figure 1 (3 days drought then re-watering) and inoculated with the Guy11 or the CL367 isolate of M. oryzae. Symptoms were taken at 7 dpi. The number of grayish lesions is indicative of the susceptibility level. [file Image_2.JPEG]

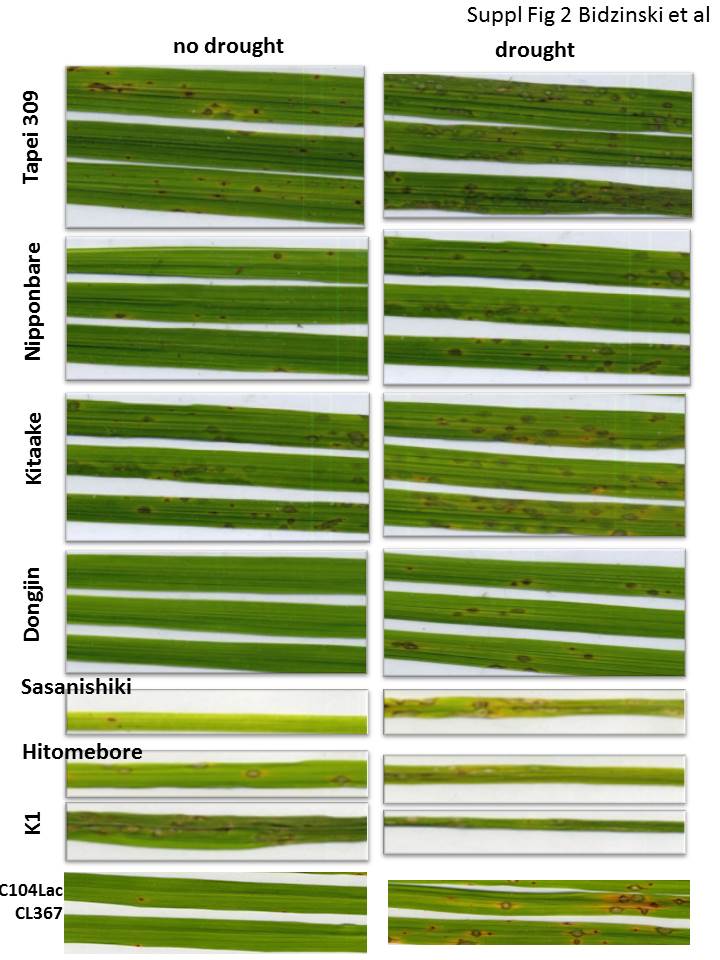

Supplement: FIGURE S3 — In planta expression of various fungal pathogenicity genes after drought. Gene expression of two biotrophic effectors fungal effectors (SLP2, BAS1), one necrotrophic effector (NEP1) and of the pathogenicity regulator MSP1 was measured by RT-qPCR during all fungal cycle in plants that experienced no stress (gray) or 3-day drought (black bars). The expression was normalized with the constitutive fungal gene MG4. The data represent the mean and SD from four replicates. A t-test was used to compare gene expression in non-stressed and 3-day stressed plants (∗∗P < 0.01; ∗P < 0.05). [file Image_3.JPEG]

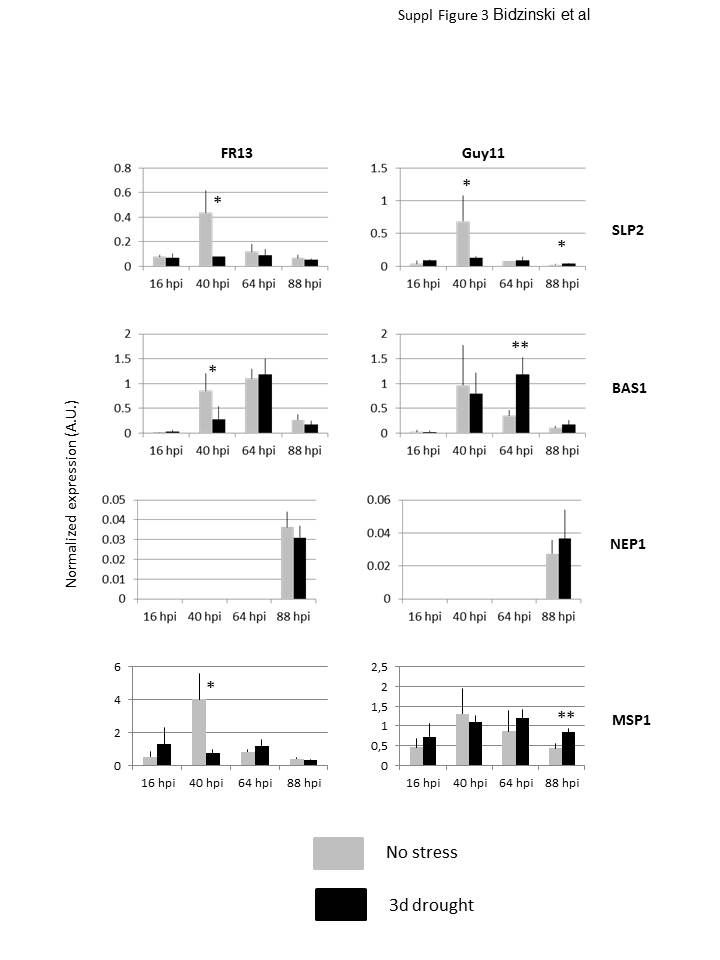

Supplement: FIGURE S4 — Plant gene expression after inoculation and drought stress. Rice gene expression was measured by RNAseq (see Materials and Methods) at 4 dpi in Nipponbare plants infected with the M. oryzae FR13 isolate. DEG analysis allowed the identification of several expression patterns and the five major ones are shown: drought-specific (D), M. oryzae specific (Mo), showing enhanced regulation (enh), non-predictable expression (not pred) and canceled (Canc). Each pattern could be subdivided depending on the induction (I) or repression (R) of the corresponding genes. For each group of genes, the average and SD values (after normalization) are shown. The letters above each condition reflect the statistics of each individual gene in each group. The number of genes in each pattern is indicated between brackets. (A) genes regulated by drought only, (B) genes regulated by infection only, (C) genes whose induction by infection is enhanced by drought, (D) genes whose expression cannot be predicted from single stresses, and (E) genes whose regulation by infection is cancelled by drought. [file Image_4.JPEG]

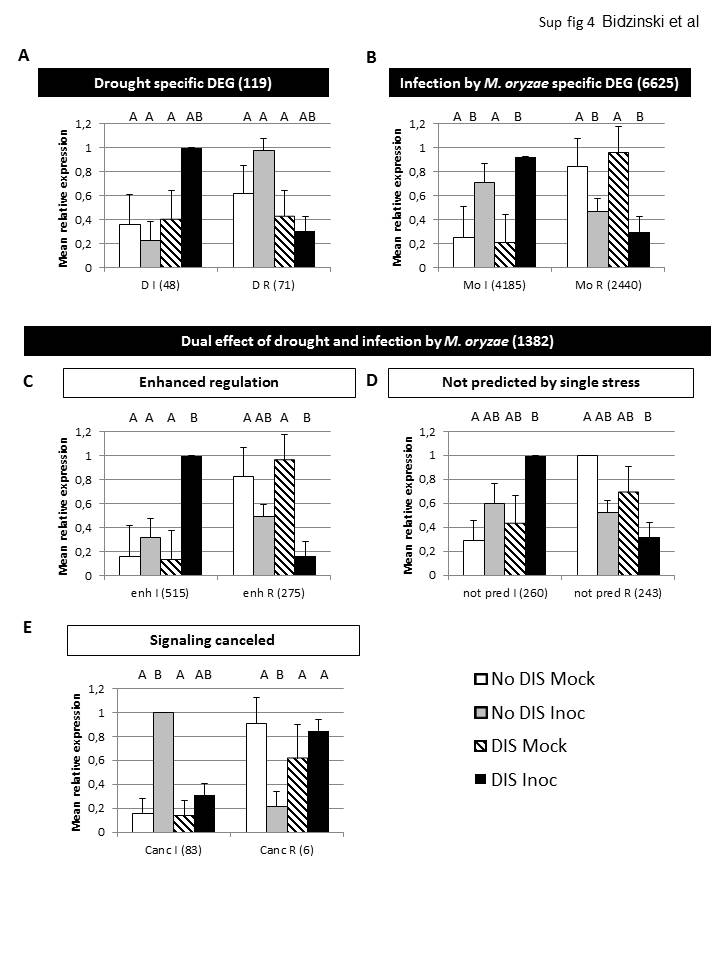

Supplement: Supplementary file 8 [file Image_5.JPEG]
