# Supplementary material for: Transcriptional Basis of Drought-Induced Susceptibility to the Rice Blast Fungus Magnaporthe oryzae
Source: Front Plant Sci. 2016 Oct 27;7:1558. doi: 10.3389/fpls.2016.01558 (PMC5081564; doi:10.3389/fpls.2016.01558)
Supplement: TABLE S1 — Primers used for RT-qPCR. [file Table_1.docx]

**Supplemental Table 1. Primers used for RT-qPCR**

| **GENE** | **Accession** | **Organism** | **Primer F** | **Primer R** |
| --- | --- | --- | --- | --- |
| NEP1 | MGG_08454 | *M. oryzae* | AAGAACGTCGGCCAGGTCTA | TCGTCGAGCCAGATGATGAC |
| SLP1 | MGG_10097 | *M. oryzae* | CCAAGCAGAGCGACAACAAC | GCGACCAGGAAGAAGGTGTC |
| SLP2 | MGG_03468 | *M. oryzae* | GGCGACAGCTTCGTTGGTAT | TTGGGCAAGTTGATGGTCTG |
| BAS1 | MGG_04795 | *M. oryzae* | ACGGTGCTTGCCACCTTTAC | CACGTCGGTGACCAACATTC |
| BAS4 | MGG_10914 | *M. oryzae* | TGCGATTGGCTCAAGAAGAA | GCCAGTAGCGCCAAGGTTAG |
| MSP1 | MGG_05344 | *M. oryzae* | CTGCTGGCAGCTCACCTACA | GGCCAATGTTGAAGCCAGAG |
| AVR-Pita1 | MGG_15370 | *M. oryzae* | CCCGTAAACAGCAGGGAAAT | CATCCCATTCGCAACCATAA |
| *OsDhn1* | LOC_Os02g44870 | *O. sativa* | AGGAGAGCCTCCTCTCCAAG | CTCCTTGAGCCCCTTCTTCT |
| *OsDREB2A* | LOC_Os01g07120 | *O. sativa* | TAAGTGGGTGGCTGAGATCC | GCAAAATTGACACGTGCTGT |
| Oshox22 | LOC_Os04g45810 | *O. sativa* | CGGACGTGTCGGTGGAG | GGGAACAAGCCCTCATCGTA |
| *OsNAC6* | LOC_Os01g66120 | *O. sativa* | GGCAAGCCATTCTAGACGAC | GCTCGCCTGAGTCAAAGTTC |
